# Increased Circulating Th17 Cells, Serum IL-17A, and IL-23 in Takayasu Arteritis

**DOI:** 10.1155/2016/7841718

**Published:** 2016-03-13

**Authors:** Durga Prasanna Misra, Smriti Chaurasia, Ramnath Misra

**Affiliations:** Department of Clinical Immunology, Sanjay Gandhi Post Graduate Institute of Medical Sciences, Rae Bareily Road, Lucknow, Uttar Pradesh 226 014, India

## Abstract

*Introduction.* Th17, *γδ*T, NK, and NKT cells in peripheral blood and serum IL-17 and IL-23 in Takayasu arteritis (TA) were measured and correlated with disease activity.* Methods.* Th17 (anti-CD3APC, CD4PECy7, and IL-17PE), NKT, NK (anti-CD3APC, CD56FITC), and *γδ*T (anti-CD3FITC and *γδ*TCRAPC) cells were enumerated by flow cytometry in peripheral blood of 30 patients with TA (ACR1990 criteria) and 20 healthy controls, serum IL-17 and IL-23 measured by ELISA. Relation with disease activity (NIH criteria, ITAS2010) was analyzed (using nonparametric tests, median with interquartile range).* Results.* Mean age of patients was 33.47 ± 11.78 years (25 females); mean symptom duration was 7.1 ± 5.3 years. 13 were not on immunosuppressants; 12 were active (ITAS2010 ≥ 4). The percentage of Th17 cells was significantly expanded in TA (patients 2.1 (1.5–3.2) versus controls 0.75 (0.32–1.2); *p* < 0.0001) with no differences in other cell populations. Serum IL-17 and IL-23 (pg/mL) in patients (6.2 (4.6–8.5) and 15 (14.9–26.5), resp.) were significantly higher (*p* < 0.001) than controls (3.9 (3.9–7.3) and undetectable median value, resp.). Subgroup analysis revealed no correlation of Th17 cells, serum IL-17, and IL-23 with disease activity or medications, nor any significant difference before and after medication.* Conclusions.* There is significant expansion of Th17 cells and elevated serum IL-17 and IL-23 levels in TA patients compared to healthy controls.

## 1. Introduction

Takayasu arteritis (TA) is a rare granulomatous large vessel vasculitis (LVV) of unknown etiology. The pathogenesis of TA has not yet been clearly elucidated; both innate and adaptive immunity are involved. Innate immune cells, gamma delta (*γδ*) T lymphocytes and natural killer cells, are increased in aortic tissue biopsies of TA [1]. Adaptive immunity also plays a role in TA peripheral blood of patients having an increased ratio of CD4^+^/CD8^+^ T cells [2]. T-helper cells producing IFN-*γ* (Th1 cells) promote granulomatous inflammation in TA. Studies in Giant Cell Arteritis (GCA), the other variant of LVV, have shown increased Th1 and Th17 (T-helper 17 cells, producing IL-17A) in peripheral blood and also elevated serum IFN-*γ* and IL-17A [3]. Recent studies have shown efficacy of IL-6 receptor blockade (tocilizumab) in refractory TA [4]. IL-6 skews naïve T-helper cells to a Th17 phenotype, maintained by IL-23 secreted by antigen presenting cells [5, 6]. Natural killer (NK) cells, natural killer T (NKT) cells, and *γδ* T cells also produce IL-17A [5, 7]. Since the role of IL-17A has not yet been clearly defined in TA, we proposed to study cell populations that are producers of IL-17A in peripheral blood, that is, Th17 cells, NK cells, NKT cells, and *γδ* T cells, and serum levels of IL-17A and IL-23 in patients with TA, and looked for their relationship with clinical disease activity.

## 2. Materials and Methods

The study was approved by the Institute Ethics Committee, SGPGIMS, Lucknow (Ethics committee number 2013-24-DM-Exp) and performed in accordance with the ethical standards laid down in the 1964 Declaration of Helsinki and its later amendments. Thirty consecutive patients fulfilling 1990 American College of Rheumatology criteria for TA [8] were included after seeking informed consent. For comparison, 20 healthy controls of similar age and sex were included after obtaining informed consent. Erythrocyte sedimentation rate (ESR) was measured by Westergren's method for all patients at time of sampling. Clinical activity was assessed using ITAS2010, ITAS-A [9], and NIH criteria [10]. 1.0 mL of heparinized whole blood was collected and flow cytometry performed immediately to enumerate cell populations under study. Serum was stored at −80°C for IL-17A and IL-23 estimation. 10 patients presented to us for the first time and were diagnosed with Takayasu arteritis (TA); hence they were not on treatment before (i.e., treatment-naïve). Clinical assessment of active disease merited starting immunosuppressive treatment. They were treated as per the standard practice at our clinic with oral weekly methotrexate (15–25 mg/week) and prednisolone (1 mg/kg/day for 6 weeks followed by gradual taper to 7.5 mg/day over 3 to 6 months). For these patients, repeat blood sampling was done after 3 months for comparison with baseline sample after receiving immunosuppressant. All analyses were done using nonparametric tests using Graph Pad Prism software version 6.0e (values represented as median with interquartile range in brackets with Mann-Whitney test used for intergroup comparison).

### 2.1. Cell Subsets Analysis by FACS

#### 2.1.1. Th17 Cells

Heparinized whole blood cultured with complete RPMI medium and 10% FCS in the ratio of 1 : 1 was activated with 50 ng/mL phorbol myristate acetate (PMA; Sigma, St. Louis, USA) and 1 *μ*g/mL ionomycin (Sigma, St. Louis, USA) for 6 hours and Golgi Plug 2 *μ*M monensin (Sigma, St. Louis, USA) was added for the last 4 four hours of activation. Cells were surface-stained with PECy7-labeled monoclonal anti-CD4 and APC-labeled monoclonal anti-CD3 antibodies (BD Biosciences, Franklin Lakes, New Jersey, USA) followed by red blood cells (RBC) lysis using FACS lysing solution (BD Biosciences, Franklin Lakes, New Jersey, USA). After washing with phosphate buffered saline (PBS), the cells were fixed using a fixation buffer (Leucoperm, ABD Serotech, San Diego, CA, USA) and washed twice with PBS (pH7.2, 0.15 M). The cells were then permeabilized with permeabilization buffer (Leucoperm, ABD Serotech, San Diego, CA, USA) and intracellularly stained with phycoerythrin- (PE-) labeled monoclonal antibodies against IL-17A. Cells were washed and resuspended in PBS.

#### 2.1.2. *γδ* T Cells

100 microlitre of heparinized whole blood was surface-stained with FITC-labeled anti-CD3 antibodies and APC-conjugated anti-*γδ* TCR (BD Bioscience, Franklin Lakes, New Jersey, USA) and lysed and washed as mentioned above.

#### 2.1.3. NK and NKT Cells

100 microlitre of heparinized whole blood was surface-stained with APC-labeled anti-CD3 and FITC-labeled anti-CD56 antibodies (BD Bioscience, Franklin Lakes, New Jersey, USA) and lysed and washed as mentioned above. In each assay, markers were drawn based on data from isotype-specific IgG antibodies.

A total of 100,000 events for Th17 cells and 50,000 events each for *γδ* T cells, NK, and NKT cells were acquired. NK cells were identified as CD3^−^CD56^+^, NKT cells were identified as CD3^+^CD56^+^ (in lymphocyte gate), *γδ* T cells were *γδ*-TCR and CD3^+^ (in CD3 gate), and CD3^+^CD4^+^ cells positive for IL-17 were identified as Th17 cells (in CD3 gate). Acquisition of cells was done on Beckman coulter flow cytometer and analyzed by Navios software.

#### 2.1.4. Serum IL-17A and IL-23

IL-17A and IL-23 were measured by ELISA as per manufacturer instructions (eBiosciences, San-Diego, USA) (sensitivity for serum IL-17A, 4 pg/mL, serum IL-23, 15 pg/mL).

## 3. Results

Demographic characteristics of patients and controls are detailed in Table 1. Both were comparable for age and gender distribution.

**Figure 1:**
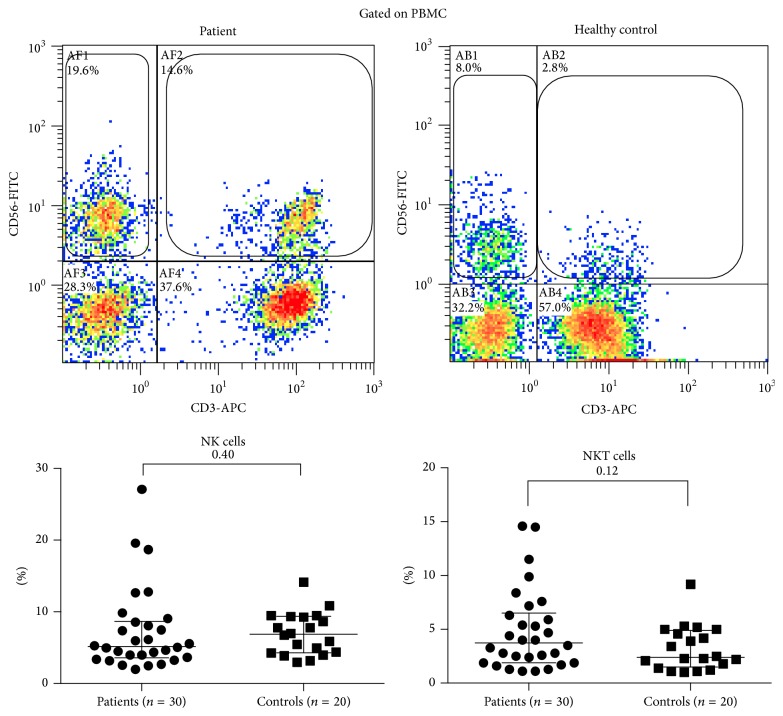
In peripheral blood of 30 patients and 20 controls, natural killer (NK) and natural killer T (NKT) cells were identified by gating on PBMCs and surface staining for CD3 and CD56. NK cells were CD3^−^CD56^+^ and NKT cells were CD3^+^CD56^+^. Bars represent median with interquartile range. *p* value (Mann-Whitney test) is mentioned in each figure.

**Figure 2:**
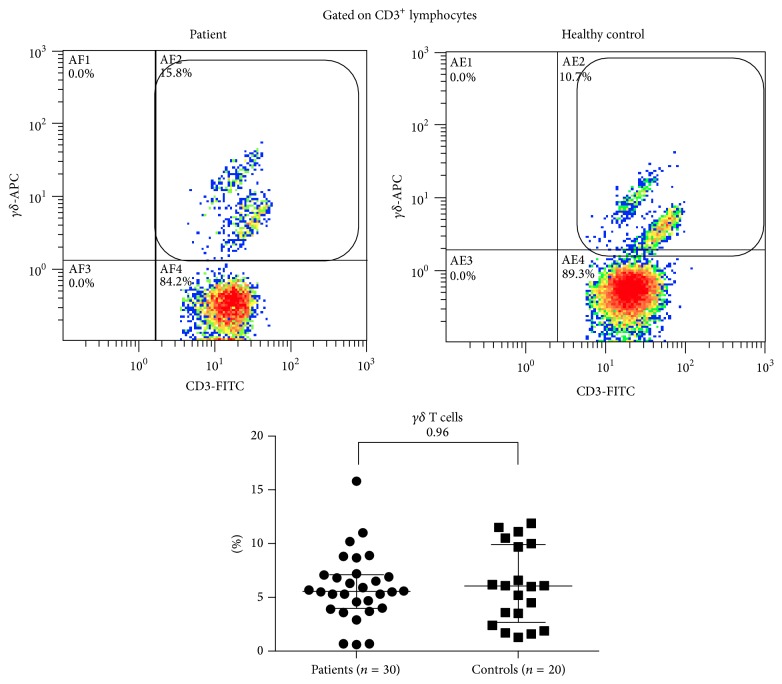
In peripheral blood of 30 patients and 20 controls, *γδ* T cells were identified by *γδ* TCR+ cells on CD3 gate. Bars represent median with interquartile range. *p* value (Mann-Whitney test) is mentioned in each figure.

**Figure 3:**
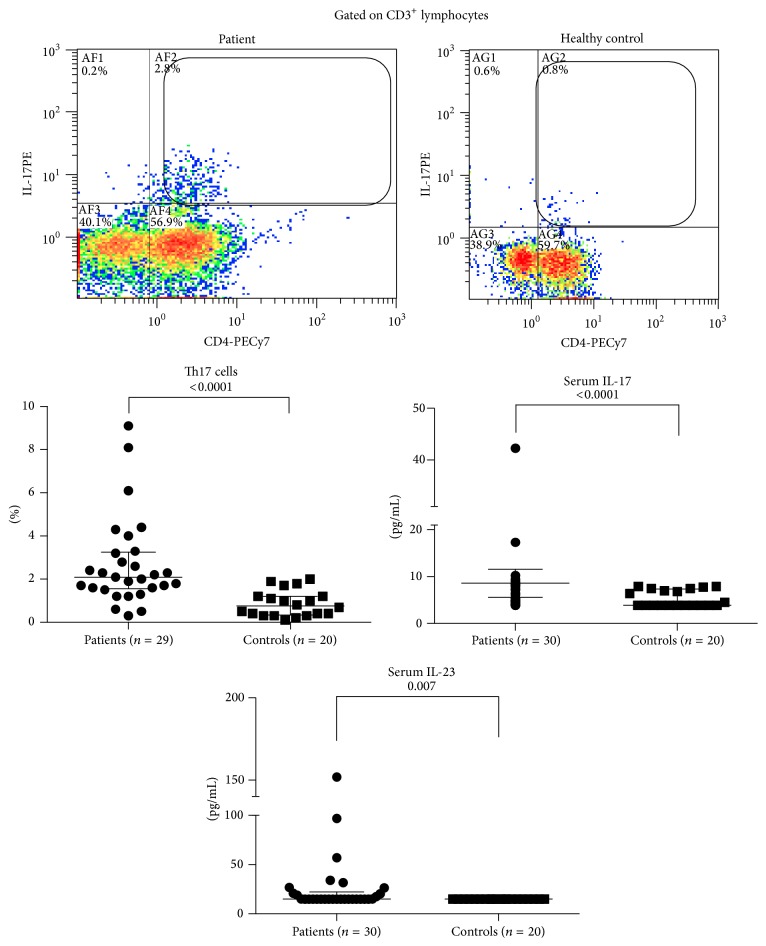
T-helper 17 (Th17) cells were measured in peripheral blood of 29 patients (could not be studied in one patient due to technical difficulties) and 20 healthy controls, after culture of whole blood for 6 hours followed by gating on CD3^+^ lymphocytes. Th17 cells were identified by surface staining for CD4 and intracellular staining for IL-17A. Serum IL-17A and IL-23 were measured by ELISA. Bars represent median with interquartile range. *p* value (Mann-Whitney test) is mentioned in each figure.

There was no significant difference in NK, NKT (Figure 1), or *γδ* T cells (Figure 2) between patients and control. However, Th17 cells were significantly expanded in patients with TA (3.1-fold higher in patients versus controls (2.69 ± 2.07 versus 0.87 ± 0.61%, resp.), *p* < 0.0001). Similarly serum IL-17A levels showed a significant elevation in patients compared to controls (1.62-fold higher in patients versus controls (8.60 ± 8.07 versus 5.32 ± 1.75 pg/mL, resp.), *p* < 0.0001). Serum IL-23 was detectable in 14 patients and in none of the controls, and the levels in patients were significantly higher than controls (1.75-fold higher in patients versus controls (26.17 ± 29.01 versus 14.9 ± 0.0 pg/mL, resp.), *p* = 0.0007) (Figure 3). Th17 cells, serum IL-17 A, or IL-23 did not correlate significantly with ESR (Pearson's correlation coefficient −0.08, −0.34, and −0.21, resp., *p* > 0.05 for all), ITAS2010 (Pearson's correlation coefficient −0.23, +0.05, and −0.25, resp., *p* > 0.05 for all), or ITAS-A (Pearson's correlation coefficient −0.25, −0.02, and −0.26, resp., *p* > 0.05 for all). There was no significant correlation demonstrable between Th17 cells and serum IL-17 (Pearson's correlation coefficient −0.11, *p* = 0.58) or IL-23 (Pearson's correlation coefficient +0.37, *p* = 0.052). Thirteen patients were active as assessed by NIH criteria, and 12 were active as assessed by ITAS2010 ≥ 4. There was no significant difference in populations of Th17 cells or serum levels of IL-17A and IL-23 in between active or inactive patients. Thirteen patients were not on immunosuppressive medications, and comparing Th17 cells and serum IL-17A and IL-23 in them versus patients on immunosuppression revealed no significant difference (Table 2).

Furthermore, we compared proportions of patients active (by NIH criteria) and on immune suppressive medications as well as ITAS2010, ITAS-A, and serum levels of IL-17A and IL-23, in subgroups of patients with Th17 cell expansion (defined as levels of Th17 cells greater than 2 SD of the mean value of controls) versus those who did not and could not demonstrate any differences between these two subgroups (Table 3).

**Figure 4:**
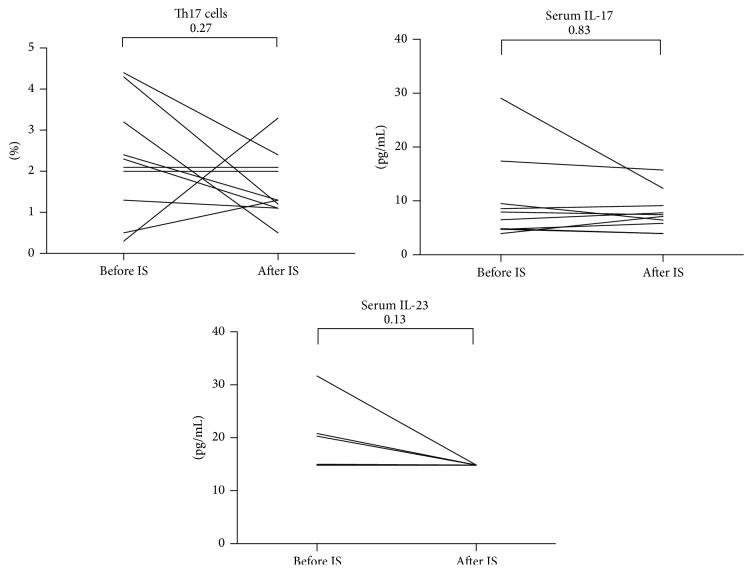
For the subgroup of 10 patients who were immunosuppressive- (IS-) naïve and were started on treatment (prednisolone and methotrexate), T-helper 17 (Th17) cells and serum IL-17A and IL-23 before and 3 months after therapy were recorded. There were no significant differences. Serum IL-23 was detectable in 3 patients prior to immunosuppression and in none of them after. *p* value (Mann-Whitney test) is mentioned in each figure.

For the 10 patients for whom we had paired samples before and 3 months after immunosuppression (prednisolone and methotrexate), there was a decline in Th17 cells following treatment. Serum IL-17A levels remained similar, and serum IL-23 was detectable in 3 patients before and none after immunosuppression. None of these differences reached statistical significance (Figure 4). However, post hoc power analysis revealed that the study was not sufficiently powered to distinguish the effect of medication on the levels of Th17 cells and serum IL-17 and IL-23 levels.

## 4. Discussion

We found an increase in serum levels of IL-17A and IL-23 in patients with TA compared with controls. The increased levels neither correlated with overall disease activity state nor became normal with immunosuppressive therapy. We also found increased expansion of Th17 cells, which produce IL-17 A, but not with NK, NKT, or gamma delta T cells (which are other producers of IL-17A) in the peripheral blood. In a subset of immunosuppressive-naïve patients, there seemed to be no differences before or after immunosuppression in Th17 cells, serum-IL-17A, or IL-23.

Our findings corroborate a recent publication by Saadoun et al. studying T cell populations and cytokines which showed increased Th17 cells in TA patients [11], which on in vitro stimulation showed increased IL-17A and IL-23 levels compared to healthy controls. However, they demonstrated higher in vitro production of IL-17A in patients with active TA (as assessed by NIH criteria) compared with those in remission, in contrast to our findings. There were no differences in Th17 cells, serum IL-17A, and IL-23 in patients on immunosuppression. Alibaz-Oner et al. explored serum levels of cytokines in patients with TA, and contrary to our findings, they failed to demonstrate an increase in serum IL-23 levels compared to healthy controls [12]. They had not studied serum IL-17A in their patients. The strength of our study was that we assessed levels of Th17 cells, serum IL-17, and IL-23 in a subgroup of patients before and after immunosuppression and could not demonstrate a difference.

In GCA, expansion of Th17 cells and increased serum IL-17 compared to controls have been demonstrated, and these levels are suppressed with corticosteroid use [3]. Interestingly, our study confirms contrary findings in TA [11], wherein the circulating Th17 cells are not suppressed with immunosuppressive medication use, including corticosteroids. This may suggest that Th17 cells in TA and GCA have different responses to corticosteroid therapy, in spite of both being classified as large vessel vasculitides.

A recent study showed increased IL-17 mRNA levels in temporal artery biopsy samples compared to controls in spite of peripheral blood IL-17 levels being similar to controls. Levels of IL-17 mRNA when elevated in the temporal artery actually predicted sustained responsiveness to glucocorticoid treatment [13]. IL-17 polymorphisms have been linked to GCA [14], and more recently in TA [15]. Studies on pathogenesis of TA are limited by the availability of aortic tissue samples [16]. However, a recent study shows expression of IL-17A in aortic tissue biopsies of active TA patients [11].

NK cells and gamma delta T cells (which are other producers of IL-17A) have been shown to be increased in vascular tissue biopsies from patients with TA, but we could not demonstrate NK cell or gamma delta T cell expansion in the peripheral blood compared to controls. The disease process in TA originates in the walls of large vessels. Levels of cytokines and cellular alterations in the peripheral blood are only a surrogate of these changes in the aortic tissue and may not reflect those at tissue level. This might explain why there was no demonstrable correlation between Th17 cells and serum levels of IL-17, or difference with respect to disease activity or medication intake. The clinical assessment of disease activity in TA is imperfect and is a work in progress [17]. Indeed aortic biopsies have shown active inflammation in patients with TA with a normal ESR [18].

Th17 expansion has also been demonstrated in variable vessel vasculitis, that is, Behcet's disease [19]. In small vessel vasculitides, Th17 skewing has been demonstrated in patients with inactive granulomatosis with polyangiitis (Wegener's) and active eosinophilic granulomatosis with polyangiitis [20]. Th17 cells remained elevated in patients with ANCA vasculitis both in active disease as well as in remission, similar to our findings in TA [21].

The exact pathogenic significance of Th17 cell expansion in TA is unclear. Aortic biopsies from patients with TA have shown neutrophil infiltrates in vascular lesions [1]. Th17 cells, by virtue of secreting IL-17A, can recruit neutrophils to sites of vascular inflammation, and IL-23 helps sustain the population of Th17 cells. Hence, corroboration with aortic biopsies is needed to further define the exact role of Th17 cells in the pathogenesis of TA.

Rare case reports of large vessel arteritis induced by Epstein-Barr virus [22] and coxsackie virus [23] have been described in literature. The etiology of TA is not yet clear, and one of the hypotheses, emerging from such case reports, is a viral infection triggering vasculitis. Th17 cells have been linked to viral persistence [24]; hence our findings of increased Th17 cells in TA could suggest viral persistence as one of the mechanisms driving TA.

Recent literature suggests efficacy of IL-6 receptor blockade (tocilizumab) in patients with difficult-to-treat TA [4]. IL-6, acting on IL-6 receptor, expressed on naïve T-helper cells, skews differentiation towards Th17 subset. Hence our finding of Th17 expansion and increased serum IL-17 in TA may have relevance explaining the efficacy of tocilizumab therapy in TA. In view of emerging Th17-IL-17 targeted therapies in spondyloarthropathies (ustekinumab, antibody to common p40 subunit of IL12 and IL-23; secukinumab, antibody to IL-17A; brodalumab, antibody to IL-17 receptor antagonist) [25], our findings of increased IL-17 and IL-23 in TA offer potential newer therapeutic approaches in refractory TA.

The limitations of our study were a relatively small sample size. However, this must be considered in view of the fact that TA is a rare large vessel vasculitis. Also, we did not measure serum CRP but instead measured ESR using a standardized technique. Lack of aortic biopsy samples to study Th17 cell and IL-17 and IL-23 mRNA expression is another limitation of our study.

To conclude, our finding of increased Th17 cells and serum IL-17A and IL-23 in TA offers newer insights into the pathogenesis of a rare large vessel vasculitis. The lack of suppression of Th17 cells in patients receiving immunosuppression, unlike in GCA, lends credence to the hypothesis that TA and GCA are pathogenetically different. Tissue level studies looking for both innate and adaptive immune cells and cytokines will provide further support to the role of Th17 cells in this disease.

## Figures and Tables

**Table 1:** Demographic details of patients and controls.

	Patients	Controls	*p* value
Total number	30	20	

Mean age	33.5 (±11.8)	34.2 (±11.3)	0.84^*∗*^

Sex (female : male)	25 : 5	13 : 7	0.137^*∗∗*^

On any immunosuppressant	17	—	—

Median prednisolone dose (mg/day)	7.5 (6.9–20) (*n* = 14)	—	—

Median oral methotrexate dose (mg/week)	17.5 (15–20) (*n* = 11)	—	—

Median azathioprine dose (mg/day)	100 (100–150) (*n* = 3)	—	—

Angiographic type (Numano's)	V – 66.7% I – 23.3% IIb - 6.7% IIa – 3.3%	—	—

^*∗*^Mann-Whitney *U* test.

^*∗∗*^ *z*-statistic.

**Table 2:** Th17 cells, serum IL-17, and IL-23 levels with respect to disease activity parameters and immunosuppressants.

Activity measured by NIH criteria			
	Active (*n* = 13)	Inactive (*n* = 17)	*p* value (Mann-Whitney)
Th17 cells (%)	2.1 (1.4–3)	2.05 (1.6–4.3) (*n* = 16^*∗*^)	0.55
IL-17A (pg/mL)	7.4 (4.7–9)	5.3 (4.4–8.5)	0.56
IL-23 (pg/mL)	14.9 (14.9–17.7)	15 (14.9–30.5)	0.07

Activity measured by ITAS2010 ≥ 4			
	Active (*n* = 12)	Inactive (*n* = 18)	*p* value (Mann-Whitney)

Th17 cells (%)	2.2 (1.4–3.1)	1.9 (1.6–4.2) (*n* = 17^*∗*^)	0.80
IL-17A (pg/mL)	7.7 (4.7–9.2)	5.4 (4.4–8.6)	0.54
IL-23 (pg/mL)	14.9 (14.9–19)	15 (14.9–28.7)	0.13

Data with respect to immunosuppressants			
	Not on immunosuppressants (*n* = 13)	On immunosuppressants^*∗∗*^ (*n* = 17)	*p* value (Mann-Whitney)

Th17 cells (%)	2 (1.3–2.8)	2.3 (1.6–3.8) (*n* = 16^*∗*^)	0.30
IL-17A (pg/mL)	6.5 (4.7–9)	6.1 (4.4–8.6)	0.85
IL-23 (pg/mL)	14.9 (14.9–20.6)	15 (14.9–26.6)	0.36

Values represented as median with interquartile range in brackets.

^*∗*^Th17 cell population could not be studied in one patient due to technical difficulties.

^*∗∗*^14 patients were on prednisolone, 11 on oral methotrexate, and 3 on azathioprine.

**Table 3:** Comparison of Th17 expanders versus the rest.

Parameter	Patients with expansion of Th17 cells (*n* = 15)	Patients without expansion of Th17 cells (*n* = 14)	*p* value
On immunosuppression	6/15	7/14	0.157
Active by Kerr's criteria	7/15	6/14	0.157
ITAS2010	1 (0–8)	1 (0–10)	0.80
ITAS-A	3 (2–10)	3.5 (1.75–11.5)	0.73
Serum IL-17A	4.96 (4.72–8.39)	7.28 (4.29–8.78)	0.48
Serum IL-23	14.9 (14.9–26.38)	14.9 (14.9–19.37)	0.52

Values represented as median with interquartile range in brackets.

Th17 expanders were defined as those patients with TA having percentage of Th17 cells greater than 2 SD of the mean value of controls.

*p* = ns for all comparisons between the two groups.
